# Voice-Evoked Color Prediction Using Deep Neural Networks in Sound–Color Synesthesia

**DOI:** 10.3390/brainsci15050520

**Published:** 2025-05-19

**Authors:** Raminta Bartulienė, Aušra Saudargienė, Karolina Reinytė, Gustavas Davidavičius, Rūta Davidavičienė, Šarūnas Ašmantas, Gailius Raškinis, Saulius Šatkauskas

**Affiliations:** 1 Faculty of Natural Sciences, Vytautas Magnus University, LT-53361 Akademija, Lithuania; raminta.bartuliene@vdu.lt (R.B.); g.davidavicius@gmail.com (G.D.); ruta.davidaviciene@alumni.vdu.lt (R.D.); sarunas.asmantas@vdu.lt (Š.A.); 2 Research Institute of Natural and Technological Sciences, Vytautas Magnus University, LT-53361 Akademija, Lithuania; 3 Department of Health Psychology, Faculty of Public Health, Lithuanian University of Health Sciences, LT-48332 Kaunas, Lithuania; ausra.saudargiene@lsmu.lt (A.S.); karolina.reinyte@lsmu.lt (K.R.); 4 Department of Informatics, Vytautas Magnus University, LT-53361 Akademija, Lithuania; gailius.raskinis@vdu.lt; 5 Neuroscience Institute, Lithuanian University of Health Sciences, LT-44307 Kaunas, Lithuania

**Keywords:** synesthesia, chromesthesia, deep neural networks, multi-layer perceptron, classification

## Abstract

**Background/Objectives:** Synesthesia is an unusual neurological condition when stimulation of one sensory modality automatically triggers an additional sensory sensation in an additional unstimulated modality. In this study, we investigated a case of sound–color synesthesia in a female with impaired vision. After confirming a positive case of synesthesia, we aimed to determine the sound features that played a key role in the subject’s sound perception and color development. **Methods:** We applied deep neural networks and a benchmark of binary logistic regression to classify blue and pink synesthetically voice-evoked color classes using 136 voice features extracted from eight study participants’ voice recordings. **Results**: The minimum Redundancy Maximum Relevance algorithm was applied to select the 20 most relevant voice features. The recognition accuracy of 0.81 was already achieved using five features, and the best results were obtained utilizing the seventeen most informative features. The deep neural network classified previously unseen voice recordings with 0.84 accuracy, 0.81 specificity, 0.86 sensitivity, and 0.85 and 0.81 F1-scores for blue and pink classes, respectively. The machine learning algorithms revealed that voice parameters, such as Mel-frequency cepstral coefficients, Chroma vectors, and sound energy, play the most significant role. **Conclusions**: Our results suggest that a person’s voice’s pitch, tone, and energy affect different color perceptions.

## 1. Introduction

Synesthesia (from Greek meaning syn—union, aithesis—sensation) is a perceptual phenomenon in which the stimulation of one type of sensory modality, the inducer, involuntarily triggers a different kind of sensory modality sensation, the concurrent [1,2]. There are many types of synesthesia that are determined by the modalities of the inducer and the concurrent [1]. The most studied forms of this phenomenon are grapheme–color synesthesia, space–sequence synesthesia, and sound–color synesthesia, also known as chromesthesia or colored hearing [3]. Grapheme–color synesthesia is characterized by a color sensation induced by viewing numbers and/or letters [4]. In space–sequence synesthesia, abstract sequences, e.g., weekdays, months, years, and the alphabet, are perceived as arranged structures in two- or three-dimensional space [5]. Other types of synesthesia include, but are not limited to, sound–touch—where hearing sounds elicits sensations in body parts [6], lexical–gustatory—where certain words are experienced as tastes [7], mirror–touch—where the observed touch of one person induces similar sensation for an individual with a synesthesia condition [1]. This study concentrates on a common form called chromesthesia—a type of synesthesia in which sounds trigger additional color sensations [8]. The prevalence rate of synesthesia is 4% with a 1:1 female/male ratio [9,10].

The neural basis of synesthesia has garnered significant attention in cognitive neuroscience, with one of the leading explanations being the cross-activation hypothesis. This theory suggests that synesthetic experiences arise from increased connectivity and interaction between brain regions involved in processing both the inducer and the concurrent percept [11,12]. For instance, in grapheme–color synesthesia, activation of the visual processing areas associated with letters (such as the fusiform gyrus) may inadvertently activate adjacent areas responsible for color perception, particularly area V4 in the visual cortex [13]. This idea is supported by neuroimaging studies that have shown increased activation in color-selective regions during synesthetic experiences, suggesting a direct link between grapheme processing and color perception [14].

Moreover, the concept of hyper binding has been proposed as a mechanism that explains how disparate sensory inputs can be integrated into a coherent perceptual experience [13]. This mechanism involves the parietal cortex, which plays a critical role in binding sensory information from different modalities. The parietal cortex may facilitate the integration of auditory and visual information, leading to the vivid and consistent experiences reported by synesthetes [15]. This is particularly evident in studies that demonstrate enhanced connectivity between visual and auditory networks in individuals with synesthesia, indicating a neural basis for the cross-modal associations they experience [16].

One widely discussed theory for brain connectivity in synesthesia is insufficient neural pruning during early brain development. Neural pruning is the process by which the brain eliminates synaptic connections that are rarely used or under-stimulated [17]. Research using neural and anatomical imaging of infants’ brains has revealed signs of increased connectivity, which may enable cross-modal perception, leading to experiences similar to that of synesthesia [18]. Typically, this excess connectivity is lost during early development in experience-based pruning; in people with synesthesia, however, this process might not happen as expected, allowing cross-sensory connections to remain [17]. The disinhibited feedback theory offers another explanation—it suggests that synesthetic experiences may result not from increased structural connectivity, but from a lack of inhibition of feedback signals that originate from higher-order associative areas and then influence sensory regions not directly activated by the initial stimulus [19].

In addition to these neural mechanisms, cognitive processes also play a significant role in synesthesia. Since many forms of synesthesia involve culturally learned elements, it is possible that synesthetic experiences develop later, particularly around the time language skills are emerging [12]. The semantic mechanisms hypothesis suggests that the associations formed in synesthesia may be influenced by higher-order cognitive processes, such as language and conceptual understanding. For example, the meaning of a grapheme may evoke a specific color based on learned associations rather than purely sensory interactions [20]. Thus, synesthesia may not solely rely on sensory pathways but also involve cognitive frameworks that shape perceptual experiences. Overall, it is important to note that some researchers suggest synesthesia may originate from a combination of existing theories, or that as research progresses, certain types of synesthesia might require distinct explanatory models rather than relying on a single unified theory [17].

While most evidence for the neural basis of synesthetic perception comes from studies on grapheme–color synesthesia [21], emerging data also point to differences in colored-hearing synesthesia. These include enhanced structural connectivity between the frontal lobe and visual and auditory association areas—individuals with colored-hearing synesthesia show distinct hemispheric patterns of white matter integrity in the inferior fronto-occipital fasciculus (IFOF), a major pathway linking sensory areas with frontal regions [21]. Increased functional connectivity has also been observed in resting-state EEG studies, particularly between the parietal lobe and auditory cortex [22]. Beyond connectivity, increased activation of specific regions is reported, such as greater V4/V8 activation during speech processing in speech–color synesthetes [23], and increased activity in the left inferior parietal cortex (IPC), which is thought to serve as a sensory integration center that couples inducer and concurrent areas via disinhibited feedback [24].

These findings suggest that cross-activation in colored-hearing synesthesia may occur when auditory input directly engages color-processing areas, such as V4/V8, or indirectly via integrative hubs like the IPC, offering a mechanistic account of the involuntary and consistent qualities of synesthetic percepts. Following these core characteristics, specifically their stability over time and automatic occurrence, the present study investigates whether specific acoustic features of human voices are systematically associated with distinct synesthetic color experiences in a case of sound–color synesthesia. To analyze and confirm synesthesia, studies use a range of neuroimaging and neurophysiological techniques, such as magnetic resonance imaging (MRI), diffusion tensor imaging (DTI), voxel-based morphometry (VBM), and electroencephalography (EEG) [12,21,25,26]. These various imaging techniques indicate increased brain activity and structural differences between synesthetes and non-synesthetes; however, they do not provide a consistent picture, clearly defined brain regions, or a shared mechanism of communication between distinct areas [26].

**Figure 1:**
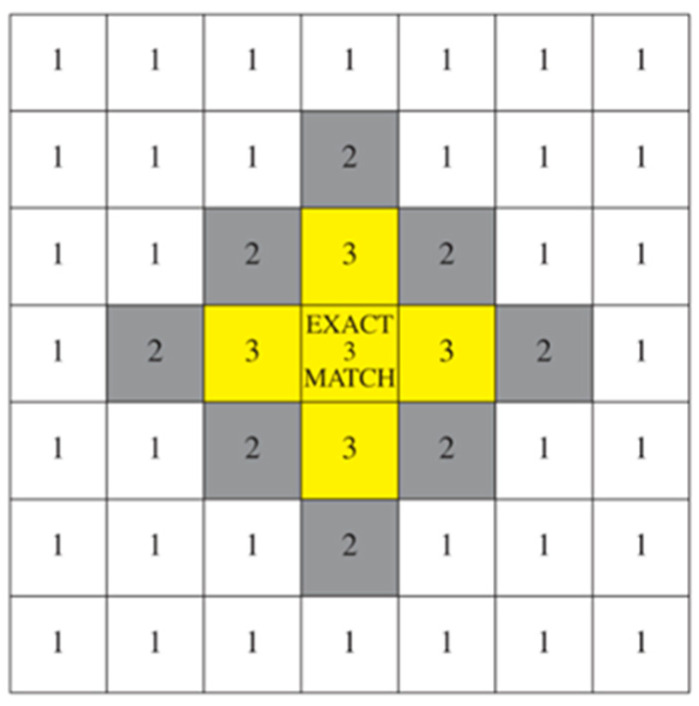
TOG scoring system (Asher et al., 2006 [29]). Exact match—3 points (boxes labeled in yellow colors), near match 1 or 2 points (boxes labeled in grey colors), and 0 points if the two swatches were not in the same color group (boxes in white colors). Each box represents a swatch on the color chart.

Since synesthetes reliably report consistent associations for specific stimuli over time, consistency is considered a hallmark of synesthesia and is thus the primary focus of most experimental validation methods [27]. To confirm the authenticity of each case, researchers typically require synesthete participants to show high levels of consistency compared to non-synesthete controls, who are asked to create and later recall similar associations (e.g., assigning colors to letters) but usually perform significantly worse [27]. The TOG [28] has become the golden standard for the reliability of self-reported synesthesia cases. It relies upon detailed descriptions of sound–color synesthesia cases. To verify whether an examined case of synesthesia is genuine, the test subject is retested after some time, and the results are compared with those of a non-synesthetic control group. It was observed that control groups generally tend to score between 20 and 38%, whereas synesthetes score between 70 and 90% [29]. In 2006, Asher et. al. [29] revised the TOG (TOG-R) by utilizing Pantone-based Cambridge Synesthesia Charts, which allowed researchers to analyze more precisely synesthetically evoked colors (Figure 1). The TOG is now one of the most used approaches in synesthesia research, likely due to the reliability of consistency test results across different types of synesthesia [27].

In summary, the methods used to study synesthesia—behavioral and neuroimaging approaches, together with TOG—provide substantial evidence supporting the authenticity of the condition [27]. Beyond identification testing, researchers have also developed questionnaires to collect detailed information about individuals’ synesthetic experiences [30].

The phenomenon of synesthesia has attracted the attention of researchers in the field of artificial intelligence. It inspired the development of machine learning models based on synesthesia principles—for instance, a 2019 study by Xing et al. [31] introduced a cross-synesthesia-aware image–music model. To build it, researchers combined emotional music and image databases, creating image–music pairs with emotional labels. In another study conducted by Xing et al. [32], deep convolutional networks were used to investigate the emotional style transfer method and match the emotion of music with the corresponding pictures. It was found that the selected methods were effective in linking the similarities between image–sound emotions.

For the study, modeling, or analysis of auditory synesthesia, a quantitative assessment of the acoustic signal is used by extracting specific features that reflect both the physical and perceptual characteristics of sound. This quantitative assessment is based on certain sound parameters that can be linked to perceived qualities such as a “whisper” or a “shout” [33]. Average Amplitude—the mean of the signal’s energy, reflecting the perceived loudness of the sound. A whisper has a low amplitude, while a shout has a high amplitude. Root Mean Square—measures the signal strength, similarly to average amplitude, but provides a more accurate representation of energy distribution [34]. Zero Crossing Rate—the number of times a signal crosses the zero-amplitude axis within a given time window. High ZCR values are characteristic of noisy, non-tonal sounds (e.g., a whisper), while low values are typical for clear tonal sounds [35].

MFCCs (Mel-frequency Cepstral Coefficients)—represent timbre and frequency information that closely corresponds to human auditory perception. Different MFCC patterns can characterize distinct voices or instruments. Delta-MFCCs—temporal changes in MFCCs that are important for dynamic voice analysis [36]. HNR (Harmonics-to-Noise Ratio)—the ratio of harmonic content to noise; a high HNR indicates a clear voice, while a low HNR reflects more noise (e.g., a whisper has a low HNR) [37]. Signal Energy—the total strength of the signal within a given time window. Energy Entropy—indicates the unevenness of energy distribution. A monotonous sound will exhibit low entropy, while a chaotic whisper will show high entropy [38].

In the auditory system, sounds are processed selectively, and their association with colors may reveal certain neural imaging characteristics. Different acoustic properties are decoded in different brain areas: The primary auditory cortex (A1) processes simple acoustic features such as frequency, amplitude, and temporal structure [39]. Secondary auditory areas (planum temporale, superior temporal gyrus) analyze more complex structures, voice timbre, and phonemic characteristics. The limbic system and prefrontal cortex are involved in the formation of emotions and subjective perceptions (e.g., “warmth” or “sharpness” of voices) [40]. Spectral entropy reflects how the brain recognizes structured and chaotic sounds [41]. Spectral roll-off reveals the perception of sharpness in sound within the auditory cortex [42].

In this study, we used a feed-forward deep neural network (DNN) to test the hypothesis that voice signal features define the voice-evoked color in a subject with a strong vision deficiency. To our best knowledge, there are no studies that used the parameters of sound signals to study sound–color synesthesia. In addition, we applied a standard statistical method, binary logistic regression (LogReg), as a benchmark method, and a more complex approach, based on deep neural networks to study the discriminant power of the voice signal parameters in identifying the color in binary prediction of the evoked color. The minimum Redundancy Maximum Relevance feature (mRMR) algorithm was applied to select the most informative features that have the greatest impact on evoked color classification. The most informative selected features were used in DNN and LogReg. The results suggest certain vocal qualities were associated with sound–color synesthesia. We identified the voice features that had the highest impact on the synesthetic color prediction.

## 2. Materials and Methods

### 2.1. The Subject with Sound–Color Synesthesia

The s synesthetic subject SB that participated in the study was a 24-year-old female who specialized in grand piano performance. At the age of three, SB had an accident that led to a loss of color vision, later progressing to severe vision impairment, leaving only the ability to perceive gray silhouettes of objects in a light environment. SB asserted that the silhouette of the person was acquiring a specific color after a few minutes of conversation. Furthermore, SB stated that the color usually remained constant over time.

SB asserted that live conversations were not required for color manifestation, and it could also develop while listening to audio recordings. Some people could evoke more than one color, up to three, but multi-colored people were not usual. Dogs, cats, and other animals had animal-specific colors.

### 2.2. Voice-Evoked Colors of the Participants and TOG Assessment

**Figure 2:**
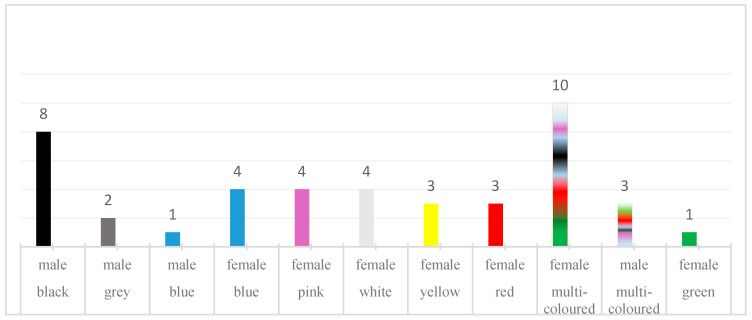
Registered single- and multi-color individuals.

First, we investigated the cases of synesthesia by conducting unstructured interviews with participants. There were 43 participants: 30 women (70%), 13 men (30%). The participants read the same excerpt from the book “The Little Prince” by Antoine de Saint-Exupery. Participants were asked to read for 5 min; therefore, the reading duration was the same. Afterward, these recordings were presented to SB for color registration (Figure 2). The group of participants was selected from students whose ages ranged from 19 to 24 years. They had no synesthetic experiences; they were healthy individuals.

Audio recordings were made using channel audio recording equipment with a 44,100 Hz sampling rate. The voice-evoked color of the silhouette was registered (Figure 2). A total of 43 participants attended this study (30 women (70%) and 13 men (30%)).

Most participants were identified as single-colored, but there were also two-colored subjects and one three-colored subject. If a participant was seen as a single-colored person, the silhouette was described as having a main color and sometimes an undertone. Multicolored people lacked a defining primary hue, and the sequence in which colors were described held no significance. SB occasionally referred to a participant’s silhouette as “mutant”, implying that the color she perceives is subject to alteration. The monochromes were exceptionally vivid. One recording was described as “finishing to mutate” and its color was erratic.

A TOG (test of genuineness) scoring system was used to assess the voice-evoked color consistency over time. TOG test has been adapted for examination of all forms of synesthesia because it does not rely on a specific stimulus set (e.g., TOG application in a case study of lexical–gustatory synesthesia) [43]. The consistency of synesthetic experiences is usually: 70–90% for synesthetes and 20–38% for non-synesthetes (Asher et al., 2006a [29]). In our study, the TOG was designed to test the consistency of synesthetic experiences over time using the same stimuli. SB was not informed about the TOG in advance. Because of the time constraints of SB, 16 randomly selected recordings of the interviews were sent to SB, and she was instructed to describe in detail the colors that manifested from the participant’s voice. Newly registered colors were compared with first encounter results to calculate a consistency score.

Asher et al. (2006a) [29] used a color scoring system in which two color swatches were compared and categorized by giving points to the swatches (Figure 1). Due to the visual impairment of SB, we could not use color swatches. Our retest evaluation relied on SB descriptions of evoked colors, categorizing the provided descriptions into matching or non-matching groups. These groups did not only account for exact matches or complete mismatches but also included responses with similar descriptions. For instance, a new undertone might be assigned in the retest (Table 1). The consistency score of SB was compared with that of a control group with similar age and education levels. In the test, the control group was instructed to select a random color and then try to memorize it. The retest was scheduled in advance and conducted after a two-week interval.

### 2.3. Voice Data Collection, Voice Feature Extraction, and Selection

Certain acoustic features of the human voice are associated with the color associations that SB experiences when listening to the voice. This means that the human voice can evoke specific color responses that are not random, but depend on objectively measurable features of the voice. Therefore, it is assumed that these features of voice signals can be used to develop machine learning models that can distinguish or even predict which color a particular voice will evoke for the synesthete. If the model can successfully distinguish colors based on voice features alone, this would confirm that there is a systematic relationship between the structure of the sound and the colors experienced during synesthesia. All recordings of the selected 8 participants had the same duration and were used for voice feature extraction.

We specifically selected recordings from a female group to avoid the potential influence of sex-specific voice differences. This choice was motivated by the larger size of the female group compared to the male group. Blue and pink colors were chosen for analysis due to their relatively frequent occurrence in our study if compared to the other colors. We included recordings from 8 female participants, with 4 representing the blue color and 4 representing the pink color. Although white color also had 4 cases, it was excluded from the analysis due to the presence of numerous comments unique to this color. For instance, SB described some instances as “shimmering” and labeled others as “mutant”—comments not found in association with any other colors. Therefore, to maintain consistency in the dataset, white was omitted from further analysis. Given the limited number of participants, we framed the task of color discrimination as a two-class problem, distinguishing between blue and pink colors.

Audio recordings were trimmed to contain 5 min of speech each, the DC component was removed, and segments of silence were eliminated using a trained binary support vector machine classifier [44]. The preparation of data for color classification was conducted in three stages: (1) short-term (primary) feature extraction, (2) medium-term feature extraction for classifier training, and (3) feature selection.

In the first stage, the audio signal was divided into adjacent, non-overlapping 50 ms frames, and 34 primary audio features, as described in Section 1, were extracted (see Table 2).

**Figure 3:**
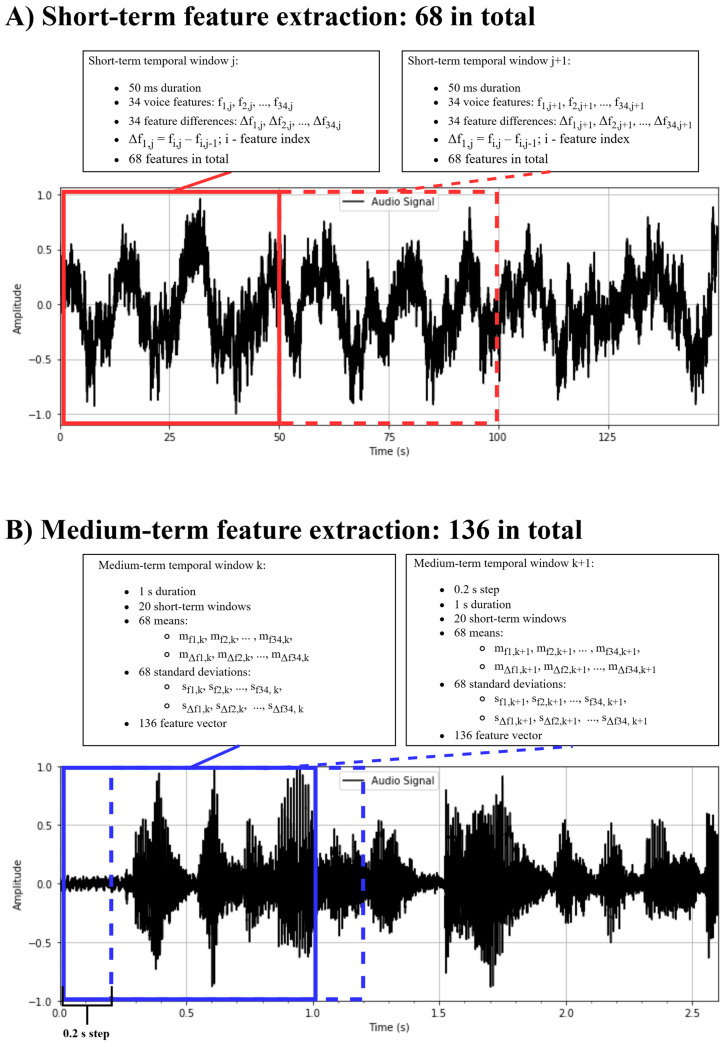
Voice feature extraction from sound WAV file segment using a moving 50 ms temporal window and a moving 1 s window. (**A**) Short-term feature extraction, 68 in total. A total of 34 voice features f_1_, f_2_, …, f_34_ were extracted using a 50 ms non-overlapping temporal window. The differences $\Delta f_{1},\Delta f_{2},..,\Delta f_{34}$ were calculated between 2 consecutive 50 ms windows, resulting in additional 34 features. For the ith feature and the jth temporal window, the difference was equal to $\Delta f_{i,j}=\Delta f_{i,j}-\Delta f_{i,j-1}$. (**B**) Medium-term feature extraction, 136 in total. Means $m_{f_{1}},m_{f_{2}},\ldots ,m_{f_{34}}$, $m_{\Delta f_{1}},m_{\Delta f_{2}},\ldots ,m_{\Delta f_{34}}$, and standard deviations $s_{f_{1}},s_{f_{2}},\ldots ,s_{f_{34}}$, $s_{\Delta f_{1}},s_{\Delta f_{2}},\ldots ,s_{\Delta f_{34}}$ were calculated across 20 consecutive 50 ms windows, resulting in 136 features for each 1 s medium-term window. The time step between medium-term windows was 0.2 s and led to 1514–1580 feature vectors per 5 min voice recording.

For each frame and each feature, the difference between two consecutive frames was calculated and appended to the primary features, resulting in a 68-dimensional short-term feature vector (see Figure 3A).

In the second stage, the audio signal was segmented into overlapping 1 s windows, shifted by 0.2 s. Each 1 s window contained 20 short-term feature vectors. From these, the mean and standard deviation of each of the 68 features were computed, yielding a 136-dimensional medium-term feature vector (see Figure 3B). Each audio clip produced approximately from 1514 to 1580 feature vectors, with slight variation due to differences in the amount of silence removed. In total, 12,374 feature vectors were extracted from the 8 selected voice recordings.

During cross-validation, all vectors derived from a particular voice recording associated with a specific color were excluded from training and used exclusively for testing, to evaluate classifier performance.

In the third stage, the minimum Redundancy Maximum Relevance (mRMR) feature selection algorithm was applied to reduce the number of input features for training logistic regression (LogReg) and deep neural networks (DNNs) [45]. The mRMR algorithm identifies the most informative and relevant features while minimizing redundancy by ranking features based on statistical measures [46]. It is capable of identifying compact subsets of functionally significant features, favoring those correlated with the output class rather than with each other. Due to its computational efficiency and effectiveness in preserving relevant model characteristics, mRMR is well suited for feature selection in machine learning tasks [47]. The resulting reduced feature set (Table 3) was used as input for both the LogReg and DNN classifiers.

**Figure 4:**
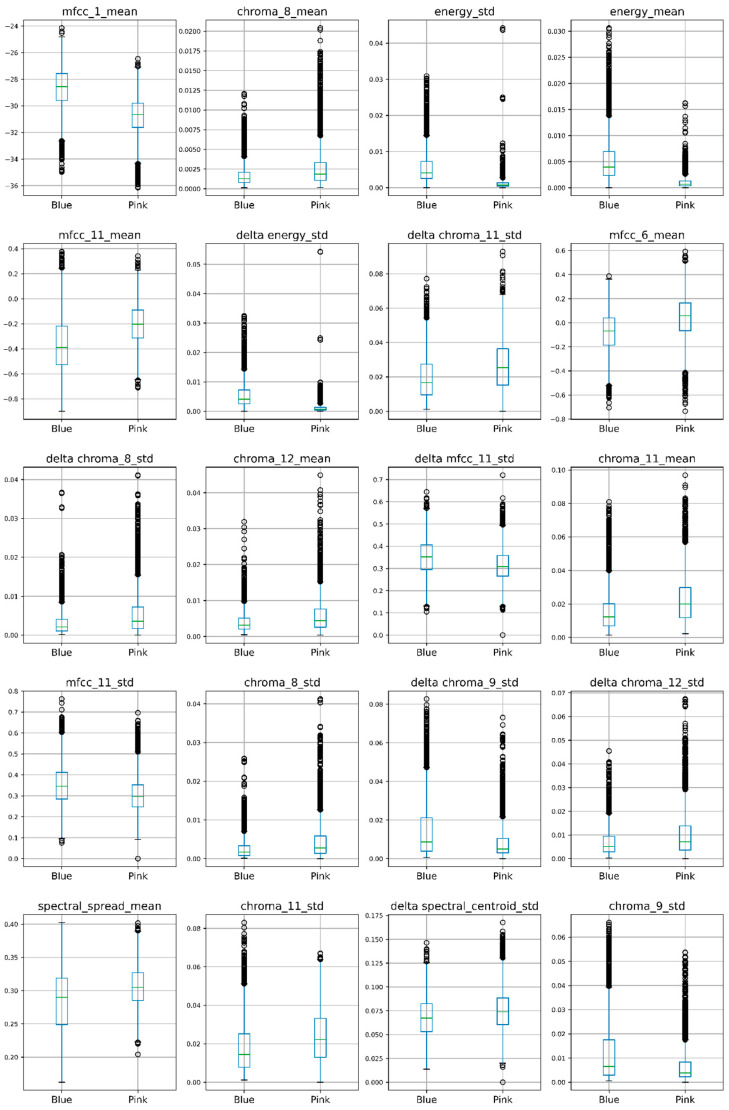
The box plots of the distributions of 20 features selected by mRMR in blue and pink color classes. Mann–Whitney U-test shows *p* < 0.01 for all features.

The mRMR feature selection algorithm was applied to reduce the total number of features used to build LogReg and train DNN [45]. The mRMR aims to identify the most informative and relevant features in a dataset while minimizing redundancy between them, using a ranking approach based on statistical measures [46]. It can find the smallest subset of functional features for machine learning tasks. mRMR tends to select features broadly related to the class (output) instead of features self-correlated with each other. Since this algorithm is computationally efficient in removing redundant features while maintaining relevant model properties in many cases, it is a good fit for feature selection in any machine-learning project [47]. The selected audio features were used as input for the LogReg and DNN methods. Dataset consisted of blue and pink female voice features (Figure 4).

### 2.4. Machine Learning Algorithms for Voice-Evoked Color Prediction

DNN is an algorithm that mimics the operational principles of the biological neuronal network [48]. DNN connects artificial neurons into a network and adjusts its weights through an error-back propagation algorithm to achieve high recognition accuracy. The back-propagation method is a procedure in which the weights of the network connections are adjusted to reduce the difference between the real output vector and the desired output vector [49].

**Figure 5:**
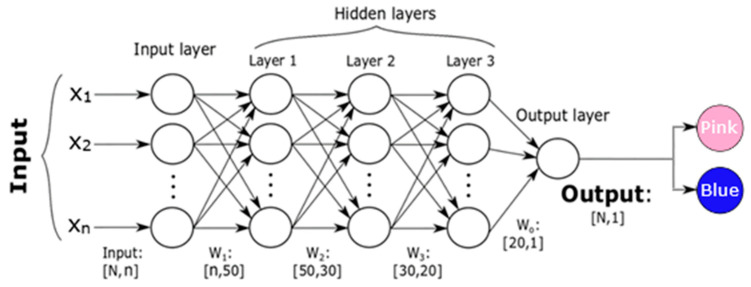
The architecture of deep neural network (DNN) used for pink and blue color classification. N—number of data samples; n—number of features; W_i_—weight matrix of the ith hidden layer x_1_, x_2_,…x_n_—a feature vector.

We used a DNN that consisted of an input layer with n input neurons (n—number of selected features), three hidden layers with 50 neurons in the first layer, 30 neurons in the second layer, 20 neurons in the third hidden layer, and 1 neuron in the output layer (Figure 5). The architecture of the neural network was chosen based on the empirical estimation of the classification accuracy. We chose the ReLU activation function for input and hidden layers and a sigmoid function in the output layer. Each layer had a 20% dropout to deactivate the neurons and reduce overfitting) [50]. The back-propagation method with binary cross-entropy function was used for the DNN training.

To assess classification accuracy, we evaluated the metrics of accuracy, sensitivity, specificity, and F1-scores. Accuracy is the percentage of predictions that were correct. F1-score is a harmonic mean of the precision and recall [51], where precision is a measure of the accuracy of a model’s positive predictions, and recall is a measure of the model’s ability to identify all positive instances correctly. The F-score value is between 0 and 1. The larger the value, the better the classification. In general classification model is evaluated by the accuracy metric, but F1-scores provide better insight into the classification of each of the classes [52]. Sensitivity is the true positive rate, which represents correctly classified pink color class samples and specificity is the true negative rate, which represents correctly classified blue color class samples.

DNNs are computationally expensive, while simpler machine learning (ML) methods perform well enough with reduced costs. We chose Logistic Regression (LogReg) to test if a simpler ML method would be sufficient to identify the voice-evoked color. LogReg is a fundamental statistical method for binary classification tasks. LogReg transforms linear regression into a framework suitable for predicting binary outcomes. By modeling the probability of a binary event occurring, LogReg offers insights into the relationship between input features and the likelihood of a specific outcome. LogReg was the most appropriate machine learning technique to compare the outcomes of DNN with, given that our objective involved binary classification. For the results to be comparable, the same metrics were used to assess the model—accuracy, F1-scores, specificity, and sensitivity.

We used *k*-fold cross-validation [53] to confirm the performance of the model. The k-fold method is often used when a small number of data is available. It is a training method that helps determine how accurately a machine learning model can classify the results of previously unseen data without sacrificing any of it [54]. When performing k-fold cross-validation, the learning set is divided into separate segments of approximately equal size. In our case, there were 4 segments for each class, resulting in a total of 16 splits. With each k-fold iteration, a different part of the data was set aside to test the model with data that were not used in training.

We had 4 representatives from each class. Twenty-five percent was excluded for data testing, one for each class. To ensure the results are not dependent on how we split the data, the K-fold was performed 16 times for all possible training–testing combinations. The model was re-initialized each time, and the training results registered are the means of all 16 splits. While the results per k-fold do vary, the total average is a good unbiased representation of the ability of the DNN to successfully classify registered colors. The remaining data were randomly shuffled and further split into training (80%) and validation (20%) data.

The Wilcoxon signed-rank test was performed to compare the classification accuracy of the DNN and LogReg models.

**Figure 6:**
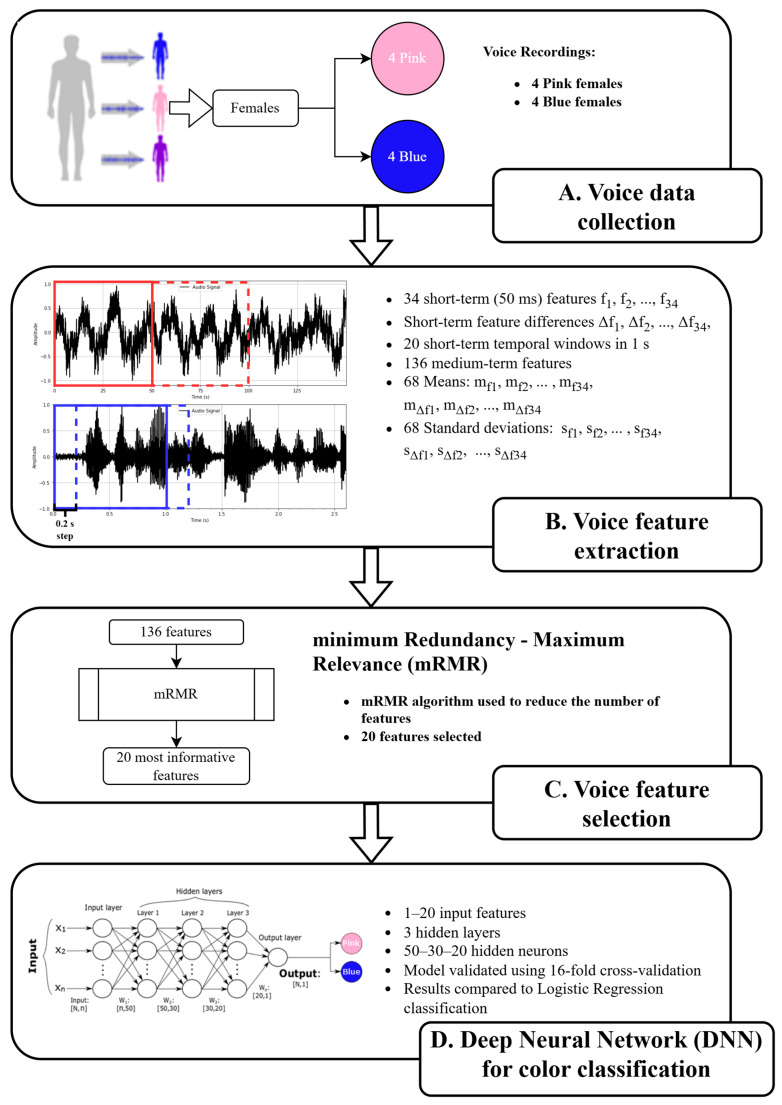
Workflow diagram of the case study of the sound-evoked color prediction (pink vs. blue) using deep neural networks and voice records of 8 participants. (**A**) The voices of 8 participants were recorded for 5 min and presented to SB for sound-evoked color synesthesia. (**B**) A total of 34 voice features f_1_, f_2_, …, f_34_ were extracted using a 50 ms non-overlapping temporal window, and the differences $\Delta f_{1},\Delta f_{2},..,\Delta f_{34}$ were calculated between 2 consecutive 50 ms windows. (**C**) The minimal Redundancy Maximal Relevance (mRMR) method was used to select the 20 most informative voice features. (**D**) A feed-forward deep neural network (DNN) with 3 hidden layers and 50, 30, and 20 neurons in each hidden layer, correspondingly, was used to classify the voice recordings into two color classes, pink and blue. The datasets included subsets of the selected features ranging from 1 to 20. A 16-fold cross-validation was applied. DNN accuracy was compared to the binary logistic regression (LogReg) performance.

The full workflow diagram of the case study of the sound-evoked color prediction using deep neural networks is shown in Figure 6.

All the selected methods were implemented using Python (version 3.11.9) programming language. We performed the audio preprocessing and feature extraction with an open-source PyAudioAnalysis Python library [44] and used the mRMR-selection library to implement the mRMR algorithm. We utilized the Keras API [55] from the TensorFlow library [56] to build and train the DNN models, and scikit-learn library [51] to build and train the LogReg model, as well as to test both the DNN and LogReg models.

## 3. Results

First, we applied the TOG test to confirm that the subject SB was synesthetic. The TOG results were compared between the synesthetic subject SB and the control subject. Second, the recorded voice signals of eight participants, evoking blue and pink colors in SB, were used to extract 134 voice features for every 1 s recording. The mRMR algorithm was applied to select the 20 most informative features. The subsets of the selected features were utilized to train DNN. The performance of DNN in the evoked color identification was compared to the accuracy of LogReg,

### 3.1. TOG Scores Based on the Recorded Voice Signals

TOG showed that SB synesthesia was genuine. The TOG accuracy score was obtained by combining color descriptions that matched completely with very similar descriptions. SB scored an accuracy of 80%, while the control subject’s score was 43.75%, confirming a positive SB case of synesthesia.

**Figure 7:**
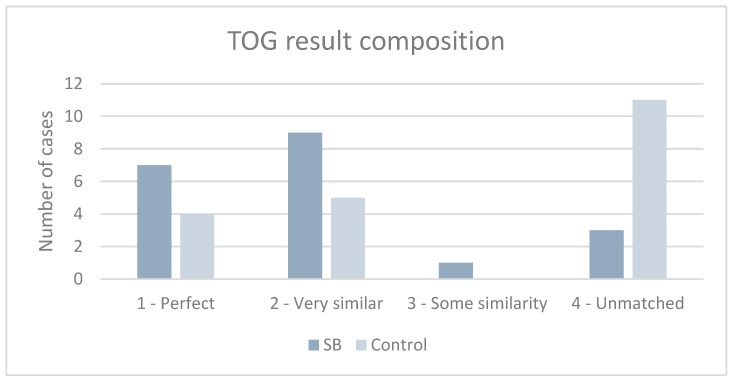
TOG result composition. Color descriptions were categorized into four groups: 1—Matched perfectly 2—Very similar 3—Unmatched but had some similarity 4—Unmatched completely.

The TOG descriptions were categorized into four groups (Figure 7). For the SB, the retest voice-evoked color of seven subjects perfectly matched the color in the first test. Nine voice-evoked color descriptions were slightly different. For example, there was an instance in which all the voice-evoked colors of a multi-colored person were listed, but the person was not identified as multi-colored. Three voice-evoked color descriptions did not match the initial color representation. One case had some similarity, specifically, only one of two colors was identified, but the person was not recognized as multi-colored. For a control case, 11 voice-evoked color descriptions did not correspond to the initial color representation, although it was allowed to use all the mnemonic tools to remember the color; even though the control subject was allowed to use all the mnemonic tools to remember the descriptions and was warned about the retest in advance.

In the subsequent part of the study, we analyzed if the participant voice signal features can be used to discriminate against the voice-evoked color in SB. We extracted and selected the most informative voice signal features and employed DNN and LogReg for voice-evoked color classification.

### 3.2. Selected Voice Features and Voice-Evoked Color Recognition Accuracy

The rank order of selected features is presented in Table 4. The top 20 features can be grouped into four groups: MFCC filters (five features), Chroma vectors (nine features), energy (five features), and spectral spread (one feature). DNN training was performed using an increasing number of selected features, starting from the first feature, and increasing the feature number.

**Figure 8:**
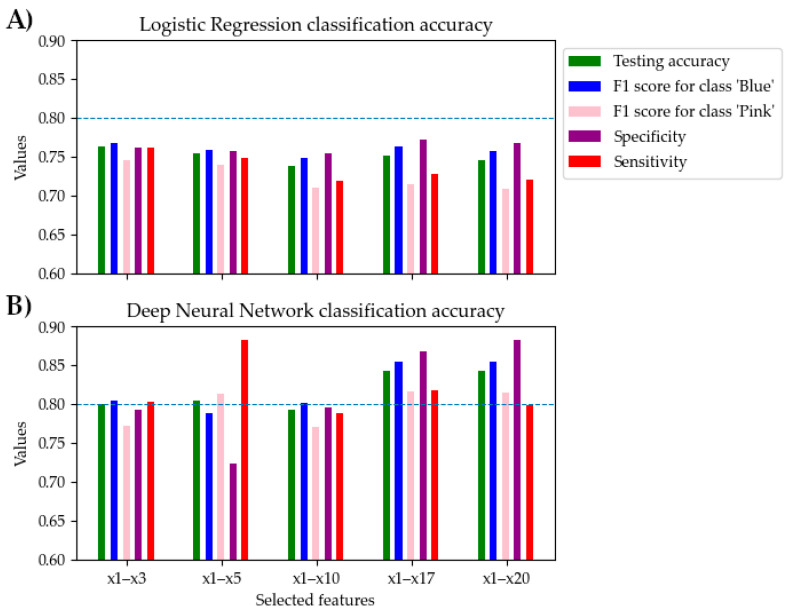
Voice-evoked color classification results using (**A**) Logistic Regression and (**B**) Deep Neural Network classifiers for different feature sets.

The results of DNN and LogReg classification performance are presented in Table 5. DNN achieved already a good discrimination accuracy (>0.8) of voice-evoked pink and blue classes using five features: mean accuracy score was 0.81 ± 0.09, mean F1-scores were 0.79 ± 0.09 for the blue color class, and 0.81 ± 0.11 for the pink color class, respectively; mean sensitivity was 0.88 ± 0.17 and mean specificity was 0.72 ± 0.11. Using fewer features is advantageous as it aids in identifying the specific voice characteristics that influence color perceptions in SB. On the other hand, employing additional features overfits the model, leading to poorer performance on unseen data. The highest DNN performance was observed utilizing 17 voice features: accuracy was 0.84 ± 0.11, F1-scores were 0.85 ± 0.08 for the blue class and 0.81 ± 0.1 for the pink class, while mean specificity and sensitivity were 0.81 ± 0.25 and 0.86 ± 0.07, respectively. The classification accuracy of the DNN outperformed the LogReg algorithm as soon as the number of features in the subset exceeded three features. The maximum mean classification accuracy LogReg achieved was 0.76 ± 0.10, mean F1-scores were 0.76 ± 0.06 for the blue class and 0.74 ± 0.14 for the pink class, sensitivity was 0.76 ± 0.22, and specificity was 0.76 ± 0.04. In summary, the DNN classifier performed better than LogReg at x_1_–x_3_ features. Figure 8 illustrates that, using the x_1_–x_5_ features, a satisfactory accuracy rate (>80%) was reached. The best result was obtained using the DNN classifier at the x_1_–x_13_ feature set.

The Wilcoxon signed-rank test indicated a statistically significant difference in the performance of DNN and LogReg, with the DNN achieving a significantly higher mean accuracy compared to the LogReg model (*p* < 0.001, T = 44031, z = 11.079). The results showed that DNN distinguished the blue color with a higher accuracy of 0.86 (±0.08) if compared to the pink color (0.82 ± 0.17); specificity and sensitivity were 0.81 (± 0.25) and 0.86 (± 0.07), respectively. The standard deviations showed the variability of the results for each data split in the cross-validation cycle. DNN classifiers resulted in a slightly higher variance in specificity and sensitivity than LogReg, e.g., 0.87 (±0.08) and 0.82 (±0.25) vs. 0.76 (±0.05) and 0.76 (±0.22), respectively, due to a higher complexity of the DNN method.

The results show the dominant voice parameters were Mel-frequency cepstral coefficients (MFCC), Chroma vectors, and sound energy. Overall, the achieved classification accuracy supports the hypothesis that color is determined by the voice parameters.

## 4. Discussion

There are numerous studies on synesthesia [57], but to date, there has not been a similar study carried out to study color synesthesia by using neural networks. In addition, synesthesia research is usually conducted with visual synesthetes. Sound color synesthetes are more involved in art [58]. In this instance, SB is a soloist, pianist, and musical personality. Therefore, it is possible that her musicality provokes synesthesia. People who have a musical pitch can categorize musical sounds without reference, while people with tone-color synesthesia can see colors when hearing music. People from both groups can perceive music better than usual.

This study attempted to analyze an unusual case of sound–color synesthesia. The hypothesis for the study was that if the silhouette colors seen by the subject are the result of different voice features, then the artificial network should be able to find these differences and classify them according to the colors induced by the synesthetic voices.

PyAudioAnalysis [44] python library was used to extract zero crossing rate, energy, entropy, spectral features, Mel-frequency cepstral coefficients, and Chroma vectors for every 50 ms window of the recordings and the deltas between separate windows were calculated. Averages and the standard deviations for each 1 s clip with a 0.2 s step for all the features and their differences were used to form the feature vector. For each voice recording, 136 voice features were extracted. In this study, we used the features extracted from blue and pink female voices that were chosen for classification. The mRMR algorithm was used to select relevant features. The feed-forward deep neural network was trained with an increasing number of features, ranked by mRMR, to determine the optimal number of relevant features. Each training iteration was performed with a 16-fold cross-validation, to eliminate any test split bias. Seventeen of the most informative voice parameters were selected, which resulted in 0.84 ± 0.09 mean testing accuracy, F1-scores of 0.85 ± 0.08 for the blue class and 0.81 ± 0.1 for the pink class, and the mean specificity and sensitivity of 0.81 ± 0.25 and 0.86 ± 0.07, respectively. The DNN allowed for better classification results than binary logistic regression, a classical machine learning algorithm. The machine learning algorithms allowed us to investigate which voice parameters play a greater role in SB sound perception: Mel-frequency cepstral coefficients (MFCCs), Chroma vectors, and sound energy. MFCCs are designed to register phonetically important characteristics of speech and sound. The Mel scale combines the pitch frequency with the actual measured frequency. The frequency is changed to find the tone that the human can hear. This feature compares bandwidth and filters that are arranged linearly in low frequencies and logarithmically in high frequencies. The Chroma vectors describe the tonal content of the audio signal. One of the main features is that it captures the harmonic and melodic characteristics of music and their similarities. Chroma functions aim to present the harmonic content of a short-term sound window. Energy shows the total size of the signal and the volume of the signal, which comes from the vibration of the sound. Our results suggest that the pitch, tone, and energy of a person’s voice all play a part in the different color elicitation for SB.

Because of the limited availability of SB, only a relatively small number of participants were included in the study. This resulted in the analysis of only the pink- and blue-evoked color categories, which consisted of four cases of pink and blue colors. Categories of other colors had from one to three cases only. The inclusion of additional colors represented by a small number of cases would have introduced considerable variability in the results of deep neural networks and statistical methods.

Mel-frequency cepstral coefficients (MFCCs), Chromatic vectors, and energy were the main factors determining the differentiation of the voices, and these features were likely more consistent or perceptually prominent in the blue voices. Various studies have shown that certain colors, especially cool ones (e.g., blue), are more common among sound–color synesthetes and are characterized by greater consistency of associations [9]. It has also been observed that these colors are more often associated with neutral or clearer emotional reactions that are less dependent on personal contexts [59]. In addition, neuroimaging studies [60] show that synesthetic experiences may be associated with the activation of areas V4/V8 of the visual cortex, suggesting that different synaptic sensitivity or levels of intersensory integration may determine different strengths of synesthetic responses. Emotional factors may play a mediating role in synaesthetic associations—studies show that colors with a clearer emotional reflection (pink—tenderness, warmth, love) may be more influenced by personal experience and at the same time less predictable [61,62]. All these factors suggest that differences in classification results reflect not only objective acoustic properties but also a complex interrelationship between sensory integration, emotional context, and individual experience. This aspect opens up wider possibilities for further research into synesthesia as a phenomenon of sensory–cognitive interaction.

Similarly to our study, previous research has shown that specific characteristics of sound, such as pitch and timbre, significantly influence the colors perceived by synesthetes. For instance, Moos et al. found that higher musical notes tend to evoke lighter colors, while lower notes are associated with darker hues. Additionally, the timbre of the sound source, whether it be a piano or a violin, can affect the richness and vibrancy of the colors experienced [63]. This suggests that the auditory properties of sounds play a crucial role in shaping the synesthetic experience, highlighting the complexity of sensory integration in synesthesia.

Nunn et al. (2002) [23], in their study, found that synesthetes who associate word sounds and colors can activate the V4/V8 area in the left hemisphere during fMRI, unlike a control group that had no changes. Therefore, it is believed that the synthesis of color and sound in people with synesthesia does not occur by chance. It is thought to be caused by aberrant cross-activation of one cortical area by another, but models differ as to whether this reflects functional or structural differences in the brains of synesthetes) [64]. The musical synesthesia color study) [65] supports the hypothesis that musical pitch tones trigger color sensations for two separate brain functions that work sequentially. First, the musical pitch is associated with its pitch class name, and then with the color. Sound emotions can play a role in color choice as a mediating factor [56,61]. Senses can also play a role (emotional mediation and sensorimotor associations can influence sense choices [66].

The role of individual differences in synesthetic experiences has also been a focal point of research. Studies have shown that the consistency and vividness of synesthetic associations can vary widely among individuals, suggesting that personal experiences and cognitive styles may influence the manifestation of synesthesia [67]. For instance, some synesthetes report deeply personal and highly idiosyncratic associations, while others may experience more standardized connections that are consistent across different contexts. This variability highlights the complexity of synesthetic experiences and the interplay between neural mechanisms and cognitive processes.

The investigation of the more diverse synesthetic colors beyond the two colors analyzed in this study could help deepen our understanding of the variability of sound-evoked color synesthesia. Furthermore, we strongly believe that future studies involving EEG recordings and analysis will help to explore the neural correlates of sound-evoked synesthesia, specifically the temporal dynamics and functional connectivity of the brain regions underlying sound–color synesthesia. This approach will enable us to link brain activity and behavioral data; here, artificial intelligence methods are the essential tools in understanding complex relations.

The implications of understanding the mechanisms of synesthesia extend beyond the phenomenon itself. Insights gained from studying synesthesia can inform broader theories of perception and cognition. For instance, the principles underlying synesthetic experiences may shed light on how the brain integrates sensory information in non-synesthetic individuals, potentially revealing universal mechanisms of perception that apply across the population [68]. Additionally, exploring synesthesia can enhance our understanding of cognitive disorders and atypical sensory processing, as the neural pathways involved in synesthesia may share similarities with those implicated in conditions such as autism spectrum disorder and schizophrenia [69].

## 5. Conclusions

By using the mRMR algorithm and feed-forward deep neural network, we found that sound–color synesthesia was related to specific voice features. Also, a feed-forward deep neural network was trained with an increasing number of features, ranked by mRMR, to determine the optimal number of relevant features. The machine learning algorithms revealed that voice parameters such as Mel-frequency cepstral coefficients (MFCCs), Chroma vectors, and sound energy play the most significant role in color perception. In addition, our results suggest that the pitch, tone, and energy of a person’s voice all play a part in a specific color perception. As research continues to unravel the intricacies of synesthesia, it promises to enrich our understanding of human perception and cognition, offering valuable insights into the nature of sensory integration and the subjective experience of reality.

## Figures and Tables

**Table 1:** Description analysis system for TOG.

**Matched**	Matched perfectly	The color of the retest matched the color of the first registration completely.
	Very similar	The main color was indicated as an undertone.
	Very similar	The main color matches, but new undertones were assigned.
	Very similar	The main color matches, but an undertone is not indicated.
	Very similar	Colors matched, but voice recording was not observed as a multi-color.
	Very similar	One color matches, but multi-colorism was not observed.
**Unmatched**	Unmatched	The colors did not match at all.
	Unmatched but had some similarity	The voice record was originally not specified as multi-colored. New color tones and new colors are assigned.

**Table 2:** The set of 34 primary features extracted from voice signals.

Feature Code	Description
zcr	Zero crossing rate: the rate of sign changes in the signal within a given frame.
energy	The sum of squared signal values, normalized by frame length.
energy_entropy	Entropy of the normalized energy across sub-frames; reflects abrupt energy changes.
spectral_centroid	The center of gravity of the spectrum
spectral_spread	The second central moment of the spectrum.
spectral_entropy	Entropy of normalized spectral energies across sub-frames.
spectral_flux	Squared difference between normalized magnitudes of successive spectral frames
spectral_rolloff	Frequency below which 90% of the total spectral energy is contained.
mfcc_1 to mfcc_13	Mel-frequency cepstral coefficients: a representation of the spectral envelope on the Mel scale.
chroma_1 to chroma_12	Chroma vector: a 12-element vector representing energy in each of the 12 pitch classes on the chromatic scale.
chroma_dev	Standard deviation of the 12 chroma coefficients

**Table 3:** Order and names of 20 selected relevant features by the mRMR algorithm.

Feature	Name
x_1_	mfcc_1_mean
x_2_	chroma_8_mean
x_3_	energy_std
x_4_	energy_mean
x_5_	mfcc_11_mean
x_6_	delta energy_std
x_7_	delta chroma_11_std
x_8_	mfcc_6_mean
x_9_	delta chroma_8_std
x_10_	chroma_12_mean
x_11_	delta mfcc_11_std
x_12_	chroma_11_mean
x_13_	mfcc_11_std
x_14_	chroma_8_std
x_15_	delta chroma_9_std
x_16_	delta chroma_12_std
x_17_	spectral_spread_mean
x_18_	chroma_11_std
x_19_	Delta spectral_centroid_std
x_20_	chroma_9_std

**Table 4:** DNN test accuracy dependence on selected features.

DNN Results						
Features	Training Accuracy	Testing Accuracy	Blue F1 Score	Pink F1 Score	Specificity	Sensitivity
x_1_	0.652 (±0.063)	0.645 (±0.098)	0.719 (±0.049)	0.489 (±0.215)	0.897 (±0.079)	0.398 (±0.252)
x_1_–x_2_	0.686 (±0.096)	0.685 (±0.117)	0.740 (±0.064)	0.560 (±0.258)	0.868 (±0.100)	0.504 (±0.303)
x_1_–x_3_	0.824 (±0.042)	0.800 (±0.115)	0.805 (±0.081)	0.772 (±0.195)	0.793 (±0.103)	0.803 (±0.270)
x_1_–x_4_	0.832 (±0.036)	0.778 (±0.106)	0.758 (±0.120)	0.773 (±0.148)	0.730 (±0.186)	0.820 (±0.232)
x_1_–x_5_	0.850 (±0.028)	0.805 (±0.093)	0.788 (±0.089)	0.813 (±0.109)	0.724 (±0.105)	0.882 (±0.172)
x_1_–x_6_	0.863 (±0.037)	0.806 (±0.105)	0.808 (±0.074)	0.789 (±0.160)	0.785 (±0.084)	0.823 (±0.249)
x_1_–x_7_	0.864 (±0.033)	0.796 (±0.118)	0.801 (±0.081)	0.771 (±0.186)	0.784 (±0.091)	0.805 (±0.274)
x_1_–x_8_	0.883 (±0.023)	0.800 (±0.130)	0.815 (±0.087)	0.763 (±0.210)	0.822 (±0.057)	0.774 (±0.285)
x_1_–x_9_	0.886 (±0.021)	0.805 (±0.122)	0.819 (±0.081)	0.771 (±0.196)	0.828 (±0.058)	0.778 (±0.274)
x_1_–x_10_	0.880 (±0.023)	0.793 (±0.122)	0.801 (±0.091)	0.770 (±0.182)	0.795 (±0.087)	0.788 (±0.253)
x_1_–x_11_	0.882 (±0.026)	0.811 (±0.119)	0.825 (±0.085)	0.786 (±0.174)	0.839 (±0.033)	0.782 (±0.238)
x_1_–x_12_	0.885 (±0.022)	0.790 (±0.123)	0.804 (±0.083)	0.749 (±0.210)	0.821 (±0.100)	0.755 (±0.289)
x_1_–x_13_	0.892 (±0.022)	0.801 (±0.108)	0.812 (±0.074)	0.775 (±0.167)	0.826 (±0.069)	0.773 (±0.238)
x_1_–x_14_	0.891 (±0.019)	0.814 (±0.112)	0.826 (±0.075)	0.785 (±0.184)	0.840 (±0.056)	0.787 (±0.247)
x_1_–x_15_	0.890 (±0.025)	0.805 (±0.105)	0.810 (±0.074)	0.788 (±0.153)	0.802 (±0.070)	0.805 (±0.229)
x_1_–x_16_	0.896 (±0.020)	0.805 (±0.112)	0.814 (±0.077)	0.782 (±0.169)	0.811 (±0.056)	0.796 (±0.245)
x_1_–x_17_	0.922 (±0.025)	0.843 (±0.114)	0.855 (±0.083)	0.816 (±0.174)	0.867 (±0.077)	0.817 (±0.252)
x_1_–x_18_	0.918 (±0.029)	0.814 (±0.145)	0.843 (±0.090)	0.741 (±0.272)	0.896 (±0.060)	0.730 (±0.338)
x_1_–x_19_	0.924 (±0.021)	0.837 (±0.113)	0.849 (±0.079)	0.810 (±0.176)	0.853 (±0.059)	0.819 (±0.256)
x_1_–x_20_	0.925 (±0.022)	0.842 (±0.108)	0.855 (±0.078)	0.814 (±0.163)	0.883 (±0.070)	0.799 (±0.238)

**Table 5:** LogReg test accuracy dependence on selected features.

LogReg Results						
Features	Training Accuracy	Testing Accuracy	Blue F1 Score	Pink F1 Score	Specificity	Sensitivity
x_1_	0.771 (±0.027)	0.761 (±0.100)	0.767 (±0.066)	0.745 (±0.149)	0.760 (±0.047)	0.759 (±0.222)
x_1_–x_2_	0.771 (±0.027)	0.762 (±0.100)	0.767 (±0.066)	0.745 (±0.149)	0.761 (±0.047)	0.760 (±0.223)
x_1_–x_3_	0.772 (±0.027)	0.763 (±0.100)	0.768 (±0.066)	0.746 (±0.149)	0.761 (±0.047)	0.761 (±0.223)
x_1_–x_4_	0.773 (±0.027)	0.763 (±0.101)	0.768 (±0.066)	0.746 (±0.149)	0.761 (±0.048)	0.762 (±0.223)
x_1_–x_5_	0.778 (±0.023)	0.754 (±0.095)	0.759 (±0.061)	0.739 (±0.141)	0.757 (±0.032)	0.748 (±0.206)
x_1_–x_6_	0.779 (±0.023)	0.755 (±0.094)	0.760 (±0.061)	0.740 (±0.141)	0.758 (±0.032)	0.749 (±0.206)
x_1_–x_7_	0.779 (±0.024)	0.753 (±0.099)	0.759 (±0.063)	0.735 (±0.150)	0.758 (±0.034)	0.745 (±0.217)
x_1_–x_8_	0.783 (±0.029)	0.736 (±0.115)	0.747 (±0.072)	0.708 (±0.183)	0.753 (±0.040)	0.717 (±0.248)
x_1_–x_9_	0.784 (±0.029)	0.738 (±0.116)	0.749 (±0.073)	0.710 (±0.184)	0.754 (±0.039)	0.719 (±0.249)
x_1_–x_10_	0.785 (±0.029)	0.738 (±0.116)	0.749 (±0.073)	0.710 (±0.184)	0.754 (±0.039)	0.719 (±0.249)
x_1_–x_11_	0.784 (±0.029)	0.734 (±0.114)	0.745 (±0.071)	0.707 (±0.182)	0.750 (±0.039)	0.715 (±0.247)
x_1_–x_12_	0.784 (±0.029)	0.734 (±0.115)	0.745 (±0.071)	0.705 (±0.184)	0.751 (±0.039)	0.714 (±0.249)
x_1_–x_13_	0.787 (±0.028)	0.734 (±0.114)	0.745 (±0.071)	0.706 (±0.182)	0.751 (±0.043)	0.714 (±0.248)
x_1_–x_14_	0.788 (±0.029)	0.734 (±0.114)	0.745 (±0.071)	0.706 (±0.182)	0.752 (±0.043)	0.714 (±0.248)
x_1_–x_15_	0.789 (±0.028)	0.735 (±0.114)	0.746 (±0.071)	0.706 (±0.184)	0.753 (±0.043)	0.714 (±0.249)
x_1_–x_16_	0.789 (±0.029)	0.734 (±0.115)	0.746 (±0.071)	0.705 (±0.185)	0.753 (±0.043)	0.713 (±0.251)
x_1_–x_17_	0.809 (±0.035)	0.752 (±0.125)	0.763 (±0.087)	0.714 (±0.208)	0.772 (±0.096)	0.728 (±0.283)
x_1_–x_18_	0.810 (±0.036)	0.750 (±0.126)	0.761 (±0.088)	0.712 (±0.210)	0.770 (±0.099)	0.726 (±0.286)
x_1_–x_19_	0.810 (±0.035)	0.747 (±0.123)	0.758 (±0.085)	0.708 (±0.208)	0.768 (±0.098)	0.722 (±0.283)
x_1_–x_20_	0.811 (±0.035)	0.746 (±0.123)	0.757 (±0.086)	0.708 (±0.208)	0.767 (±0.101)	0.721 (±0.283)

## Data Availability

The data that support the findings of this study are available upon request due to privacy.
